# Emergence of SARS-CoV-2 B.1.1.7 Lineage at Outpatient Testing Site, Berlin, Germany, January–March 2021

**DOI:** 10.3201/eid2707.210845

**Published:** 2021-07

**Authors:** Welmoed van Loon, Heike Rössig, Susen Burock, Jörg Hofmann, Julian Bernhard, Elisabeth Linzbach, Domenika Pettenkofer, Christian Schönfeld, Maximilian Gertler, Joachim Seybold, Tobias Kurth, Frank P. Mockenhaupt

**Affiliations:** Charité–Universitätsmedizin Berlin, Berlin, Germany (W. van Loon, H. Rössig, S. Burock, J. Bernhard, E. Linzbach, D. Pettenkofer, C. Schönfeld, M. Gertler, J. Seybold, T. Kurth, F.P. Mockenhaupt);; Labor Berlin–Charité Vivantes GmbH, Berlin (J. Hofmann)

**Keywords:** COVID-19, coronavirus disease, SARS-CoV-2, severe acute respiratory syndrome coronavirus 2, viruses, respiratory infections, zoonoses, B.1.1.7, Berlin, Germany, viral load

## Abstract

Within 5 weeks in 2021, B.1.1.7 became the dominant severe acute respiratory syndrome coronavirus 2 lineage at an outpatient testing site in Berlin, Germany. Compared with outpatients with wild-type virus infection, patients with B.1.1.7 had similar cycle threshold values, more frequent sore throat and travel history, and less frequent anosmia/ageusia.

Severe acute respiratory syndrome coronavirus 2 (SARS-CoV-2) B.1.1.7 lineage (variant of concern [VOC] 202012/01 or 20I/501Y.V1) likely emerged during autumn 2020 in the United Kingdom and quickly became dominant there (E. Volz et al., unpub. data, https://www.medrxiv.org/content/10.1101/2020.12.30.20249034v2). B.1.1.7 carries multiple mutations and deletions, including 501Y and deletion ΔH69/ΔV70 (del69–70) in the spike protein. B.1.1.7 reportedly exhibits greater transmissibility and fatality in the community than non-VOC lineages (hereafter referred to as wild-type virus) (*1*; E. Volz et al., unpub. data). However, increased deaths were not seen in hospitalized patients (*2*).

The first patient infected with B.1.1.7 at the outpatient SARS-CoV-2 testing site of Charité–Universitätsmedizin Berlin was identified on January 18, 2021. We describe lineage prevalence over time and demographic and clinical characteristics in outpatients with B.1.1.7 or wild-type virus who sought care during January–March 2021. Ethics approval was obtained from Charité’s Institutional Review Board (EA4/083/20).

## The Study

Details of the testing site have been described (*3*). Physicians interviewed patients about demographics, medical history, and symptoms. If indicated, a combined oro-nasopharyngeal swab specimen was collected. Specimens were tested by using the cobas 6800/8800 assay (Roche Diagnostics, https://diagnostics.roche.com), targeting open reading frame 1ab and the envelope gene (*4*). All positive samples were typed for the N501Y and del69–70 polymorphisms by melting curve analysis. Variants including both polymorphisms were considered B.1.1.7.

**Figure 1:**
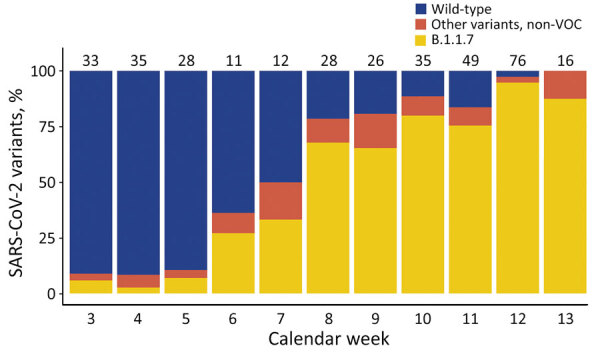
Proportion of SARS-CoV-2 lineages at the Charité–Universitätsmedizin Berlin testing site, Berlin, Germany, January–March 2021. The numbers on top of the bars indicate the total number of positive SARS-CoV-2 tests. Six (partially) vaccinated outpatients are included for completeness. Note that calendar week 13 only includes 1 day (March 29). SARS-CoV-2, severe acute respiratory syndrome coronavirus 2; VOC, variant of concern.

During January 18–March 29, 2021, a total of 349 SARS-CoV-2–positive patients were seen at the testing site, and the proportion of B.1.1.7 increased from 2% to >90% (Figure 1). In total, 35.8% (125/349) of samples belonged to the wild-type lineage, 57.0% (199/349) were B.1.1.7, and 7.2% (25/349) were other non–wild-type variants (non-VOCs).

Six patients previously had received >1 SARS-CoV-2 vaccinations; all were infected with B.1.1.7 but were excluded from analysis because vaccination could interfere with viral dynamics and clinical manifestation (Appendix Table). We excluded patients carrying lineages other than wild-type or B.1.1.7. Half of the patients were female (49%); mean age was 36 (SD +15) years. Almost all (97%) reported symptoms. Median symptom duration until testing was 3 (interquartile range 2–4) days. Symptoms were fatigue (72%), headache (69%), and muscle ache (60%). Fifteen percent reported travel outside Berlin in the previous 14 days, and half (49%) reported contact with a SARS-CoV-2–positive person (Table).

**Table:** Characteristics of severe acute respiratory syndrome coronavirus 2–positive outpatients attending the Charité–Universitätsmedizin Berlin testing site, by lineages, Germany, January–March 2021*

Characteristic	Wild-type lineage	B.1.1.7 variant	OR (95% CI)
Total no.	125	193	NA
Sex			
M	69 (55.2)	94 (49.0)	
F	56 (44.8)	98 (51.0)	1.3 (0.8–2.0)
Mean age, y (±SD)	36.6 (±13.8)	34.8 (±15.9)	1.8 (−1.5 to 5.1)†
Any symptoms	122 (97.6)	186 (96.4)	0.7 (0.2–2.6)
Self-reported fever in previous 48 h	48 (38.4)	82 (42.5)	1.2 (0.8–1.9)
Median self-reported temperature in case of fever, °C (±SD)	38.3 (±0.6)	38.2 (±0.7)	0.1 (−0.2 to 0.4)§
Shortness of breath	12 (9.6)	26 (13.5)	1.5 (0.7–3.0)
Fatigue	92 (73.6)	138 (71.5)	0.9 (0.5–1.5)
Chest pain	3 (2.4)	2 (1.0)	0.4 (0.1–2.6)
Diarrhea	19 (15.2)	24 (12.4)	0.8 (0.4–1.5)
Anosmia or ageusia (loss of smell or taste)	47 (37.6)	46 (23.8)	0.5 (0.3–0.9)
Muscle aches	75 (60.0)	116 (60.1)	1.0 (0.6–1.6)
Sore throat	52 (41.6)	104 (53.9)	1.6 (1.0–2.6)
Cough	61 (48.8)	98 (50.8)	1.1 (0.7–1.7)
Headache	86 (68.8)	133 (68.9)	1.0 (0.6–1.6)
Chills	44 (35.2)	67 (34.7)	1.0 (0.6–1.6)
Rhinorrhea	76 (60.8)	102 (52.8)	0.7 (0.5–1.1)
Median duration of symptoms upon test, d (25%–75% quantile)	3.0 (2.0–4.8)	3.0 (2.0–4.0)	0.0 (−1.0 to 0.0)§
Contact with person with confirmed SARS-CoV-2 infection	60 (48.0)	97 (50.3)	1.1 (0.7–1.7)
Median time between contact with person with confirmed SARS-CoV-2 infection and test, d (25%–75% quantile)	4.0 (1.2–7.0)	4.0 (1.0–6.0)	0.0 (−1.5 to 3.0)‡
Travel outside Berlin region in previous 14 d	12 (9.6)	36 (18.7)	2.2 (1.1–4.3)
Median C_t_ value (25%–75% quantile)	20.2 (17.4–24.1)	20.1 (17.1–22.8)	0.1 (−0.9 to 1.6)‡
Symptom duration <7 d, no. patients (median C_t_ value [25%–75% quantile])	113 (19.9 [17.4–23.5])	171 (19.5 [16.6–22.6])	0.4 (−1.0 to 1.7)‡
Symptom duration >7 d, no. patients (median C_t_ value [25%–75% quantile])	6 (30.1 [26.1–31.3])	11 (26.2 [21.4–31.2])	3.9 (−5.6 to 10.0)‡

*Values are no. (%) except as indicated. C_t_, cycle threshold; NA, not applicable; OR, odds ratio; SARS-CoV-2, severe acute respiratory syndrome coronavirus 2. †Difference in means (95% CI). ‡Difference in medians (95% CI). The 95% CI for difference in medians was computed by a percentile bootstrap with 1,000 replications.

Most assessed characteristics did not substantially differ between patients with wild-type or B.1.1.7, including age, sex, leading symptoms, symptom duration, contact with a SARS-CoV-2–positive person, and time passed since contact (Table). However, B.1.1.7 patients had traveled more frequently than those with the wild-type strain (19% vs. 10%). Patients with B.1.1.7 more often reported sore throat than did patients with wild-type virus (54% vs. 42%) but less frequently reported anosmia or ageusia (24% vs. 38%) (Table).

**Figure 2:**
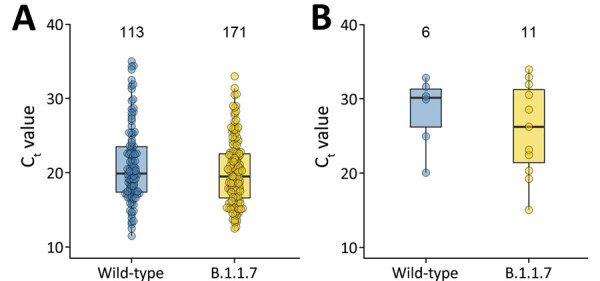
Comparison of median C_t_ values in severe acute respiratory syndrome coronavirus 2 wild-type and B.1.1.7 lineage by symptom duration, Berlin, Germany, January–March 2021. A) Symptom duration <7 days. B) Symptom duration >7 days. The boxplots indicate medians (center) and 25th (top) and 75th (bottom) percentiles (i.e., quartile [Q] 1 and Q3). The upper whiskers reach the largest value with a maximum Q3 +1.5 interquartile range. The lower whiskers reach the smallest value with a minimum Q1 –1.5 interquartile range. The numbers on top of the boxplots indicate the total number of observations included in the comparison. C_t_, cycle threshold.

We observed no difference in cycle threshold (C_t_) values for the envelope gene target between B.1.1.7 and wild-type samples (median 20.2 vs. 20.1) (Table). In patients reporting a symptom duration of >7 days, C_t_ values appeared to be lower for those with B.1.1.7 (26.2 vs. 30.1 for wild-type), but the difference was not significant (p = 0.7 by Mann–Whitney U test) (Table; Figure 2).

Finally, we explored which combination of variables in our dataset best described B.1.1.7 in a logistic regression applying a backward stepwise selection on the basis of the Akaike information criterion (Appendix). This work identified the best set of associated factors as the absence of anosmia or ageusia (p = 0.01), longer symptom duration (p = 0.02), sore throat (p = 0.05), lower C_t_ value (p = 0.07), travel in the previous 14 days (p = 0.08), lower age (p = 0.09), and absence of rhinorrhea (p = 0.12). We then used the bootstrap technique to repeat the variable selection in 1,000 replicated datasets and evaluated how often these variables were selected with the backward selection. This analysis resulted in anosmia or ageusia, 89%; symptom duration, 78%; travel, 73%; sore throat, 68%; C_t_ value, 66%; age, 56%; and rhinorrhea, 51%. Absence of anosmia or ageusia, longer symptom duration, and travel were selected most often, indicating their association with B.1.1.7 infection. We performed all analyses in R version 3.6.3 (https://cran.r-project.org).

## Conclusions

The first B.1.1.7 case in Germany was recorded in late December 2020 (*5*). At our testing site, B.1.1.7 was observed 3 weeks later and replaced wild-type virus as the dominant strain within just 5 weeks. The rapid emergence and dominance of this lineage likely results from its increased transmissibility (E. Volz et al., unpub. data), which is potentially caused by spike protein polymorphisms (including 501Y) conferring enhanced mucosal binding (*6*); 681H, near a region vital for transmission (T.P. Peacock et al., unpub. data, https://doi.org/10.1101/2020.09.30.318311); and deletion 69–70, linked to immune escape (*7*). Viral replication in vitro does not differ from earlier strains (J.C. Brown et al., unpub. data, https://doi.org/10.1101/2021.02.24.432576).

Compared to the wild-type virus, B.1.1.7 is reportedly associated with more deaths in nonhospitalized patients but not in inpatients (*1*,*2*). In our young outpatient study population, we did not observe major, lineage-dependent differences in leading symptoms. Nevertheless, anosmia and ageusia, among the most specific coronavirus disease symptoms (*3*), were less common in patients with B.1.1.7., whereas sore throat was more common. A survey in the United Kingdom revealed patients with B.1.1.7 experienced anosmia and ageusia less frequently but more frequently experienced sore throat, cough, fatigue, myalgia, and fever (*8*). In contrast, no associations between SARS-CoV-2 B.1.1.7 and self-reported symptoms, disease duration, or hospital admissions were seen in another UK study (*9*). The main factors for B.1.1.7 infection prediction in our study appeared to be lack of anosmia or ageusia and longer symptom duration, in addition to recent travel. The association with recent travel at the time of B.1.1.7 spread is probably no longer relevant because B.1.1.7 is now the most common variant in Berlin and Germany.

With regard to C_t_ values, one study observed similar figures in patients with B.1.1.7 and wild-type lineages; however, a longer duration of PCR–positivity with B.1.1.7 was suggestive by repeated sampling over time (S.M. Kissler et al., unpub. data, https://doi.org/10.1101/2021.02.16.21251535). Likewise, longer persistence has been observed for B.1.1.7 (*10*), but lower C_t_ values (indicating higher viral load) have been observed compared to wild-type samples. Lower C_t_ values were also seen in other studies on population (*11*) and inpatient levels (*2*). In our cross-sectional assessment of recently ill outpatients, we did not observe such differences. Still, increased transmissibility may result from the variant’s prolonged excretion (*10*; S.M. Kissler et al., unpub. data). Test timing appears crucial for interpretation of C_t_ values. Outpatients are commonly tested earlier than inpatients. The combination of prolonged viral shedding with different test timing might explain increased viral load in B.1.1.7 samples in inpatients but not in recently ill outpatients. In outpatients with ≥7 days symptom duration, C_t_ values in B.1.1.7 samples were suggestively reduced. However, comparison groups were small. Our data enabled detection of a maximum effect size on overall C_t_ values, which corresponds to B.1.1.7 C_t_ values being 1.5 units below or 1.0 above those for wild-type virus.

The main limitation of our study was its limited subgroup sizes, which reduced the likelihood of detecting differences between rare characteristics. Other limitations are the 1-time assessment, subjective symptom duration, and the variable manifestation of SARS-CoV-2 infection (*3*). Strengths include standardized procedures conducted by trained medical staff and its prospective nature evaluating patient groups during the same period, reducing the likelihood of biases because of temporal effects.

In summary, SARS-CoV-2 VOC B.1.1.7 is now the dominant lineage in Berlin. In outpatients, no major difference in clinical manifestations has been observed.

Appendix Additional information about the emergence of SARS-CoV-2 B.1.1.7 lineage at outpatient testing site, Berlin, Germany, January–March 2021
