# Supplementary material for: Emergence of SARS-CoV-2 B.1.1.7 Lineage at Outpatient Testing Site, Berlin, Germany, January–March 2021
Source: Emerg Infect Dis. 2021 Jul;27(7):1931–4. doi: 10.3201/eid2707.210845 (PMC8237906; doi:10.3201/eid2707.210845)
Supplement: Appendix — Additional information about the emergence of SARS-CoV-2 B.1.1.7 lineage at outpatient testing site, Berlin, Germany, January–March 2021 [file 21-0845-Techapp-s1.pdf]

Article DOI: https://doi.org/10.3201/eid2707.210845

Emergence of SARS-CoV-2 B.1.1.7 Lineage at Outpatient Testing Site, Berlin, Germany, January–March 2021

Appendix

Methods for Backward Stepwise Selection Procedure

We explored which combination of variables in our dataset best described the severe acute respiratory syndrome coronavirus 2 (SARS-CoV-2) B.1.1.7 variant in a logistic regression applying backward stepwise selection by Akaike information criterion (AIC). The following variables were included for this process: virus variant (wildtype or B.1.1.7), cycle threshold (C_t) value, sex (male or female), age (in years), symptom duration (in days), self-reported fever (no or yes), short of breath (no or yes), fatigue (no or yes), chest pain (no or yes), diarrhea (no or yes), anosmia/ageusia (no or yes), muscle ache (no or yes), sore throat (no or yes), cough (no or yes), headache (no or yes), chills (no or yes), rhinorrhea (no or yes), contact with a person with confirmed SARS-CoV-2 infection (no or yes), travel outside Berlin in the last 14 days (no or yes). Observations with missing data (e.g., for those without symptoms, no data for symptom duration was available) were excluded.

A full binomial logistic regression was fit, with virus variant as outcome variable. The full model was used for backward selection by AIC (R function stepAIC from the MASS package in R). For the bootstrap analysis, the same process was repeated on 1,000 resampled datasets (R function boot.stepAIC from the bootStepAIC package in R). In short, the following process was repeated 1,000 times: a new dataset is simulated by resampling with replacement; the full binomial logistic regression model is fit; the backward stepwise selection by AIC is done on the new full model. Among the 1,000 resulting selected models, the number of times is counted that each variable was selected.

All analyses were done in R version 3.6.3, and the code is available upon request from the corresponding author.

Appendix Table. Characteristics of non-vaccinated and vaccinated patients infected with severe acute respiratory syndrome coronavirus 2 B.1.1.7 lineage, Berlin, Germany, January–March 2021*

Characteristic	B.1.1.7 variant, without vaccination	B.1.1.7 variant, vaccinated
Total, no.	193	6
Sex, no. (%)		
M	94 (49.0)	2 (33.3)
F	98 (51.0)	4 (66.7)
Mean age, y (SD)	34.8 (15.9)	31.7 (9.2)
Any symptoms, no. (%)	186 (96.4)	6 (100)
Self-reported fever in previous 48 h	82 (42.5)	3 (50)
Median self-reported temperature in case of fever, no. patients (°C [SD])	43 (38.2 [0.7])	1 (38.2 [-])
Shortness of breath	26 (13.5)	0 (0)
Fatigue	138 (71.5)	5 (83.3)
Chest pain	2 (1.0)	0 (0)
Diarrhea	24 (12.4)	0 (0)
Anosmia or ageusia (loss of smell or taste)	46 (23.8)	2 (33.3)
Muscle aches	116 (60.1)	1 (16.7)
Sore throat	104 (53.9)	2 (33.3)
Cough	98 (50.8)	2 (33.3)
Headache	133 (68.9)	2 (33.3)
Chills	67 (34.7)	2 (33.3)
Rhinorrhea	102 (52.8)	4 (66.7)
Median duration of symptoms upon test, no. patients (d [25%, 75% quantile])	186 (3.0 [2.0, 4.0])	6 (1.0 [1.0, 1.8])
Contact with person with confirmed SARS-CoV-2 infection, no. (%)	97 (50.3)	1 (16.7)
Median time between contact with person with confirmed SARS-CoV-2 infection and test, no. patients (d [25%, 75% quantile])	96 (4.0 [1.0, 6.0])	1 (8.0 [-])
Traveled outside Berlin region in previous 14 d, no. (%)	36 (18.7)	0 (0)
Median C_t value (25%, 75% quantile)	20.1 (17.1, 22.8)	19.0 (17.4, 22.5)

*Five vaccinated patients received their first dose AZD1222 (Oxford-AstraZeneca) 4–24 days before their positive SARS-CoV-2 test; 1 received the second BNT162b2 (Pfizer/BioNTech) dose 66 days before the positive test. The 95% CI for the difference in medians was computed by a percentile bootstrap with 1,000 replications.
